# Subclinical atrial fibrillation prediction based on deep learning and strain analysis using echocardiography

**DOI:** 10.1007/s11517-025-03385-z

**Published:** 2025-05-31

**Authors:** Sung-Hao Huang, Ying-Chi Lin, Ling Chen, Sayan Unankard, Vincent S. Tseng, Hsuan-Ming Tsao, Gau-Jun Tang

**Affiliations:** 1 Division of Cardiology, Department of Internal Medicine, National Yang Ming Chiao Tung University Hospital, Yilan, Taiwan; 2 Institute of Hospital and Health Care Administration, National Yang Ming Chiao Tung University, No. 155, Sec. 2, Linong St., Beitou Dist, Taipei, Taiwan; 3 Information Technology Division, Faculty of Science, Maejo University, Chiang Mai, Thailand; 4 Department of Computer Science, National Yang Ming Chiao Tung University, Hsinchu, Taiwan; 5 School of Medicine, National Yang Ming Chiao Tung University, Taipei, Taiwan

**Keywords:** Deep learning, Left atrial segmentation, Left atrial strain, Atrial high-rate episodes

## Abstract

Subclinical atrial fibrillation (SCAF), also known as atrial high-rate episodes (AHREs), refers to asymptomatic heart rate elevations associated with increased risks of atrial fibrillation and cardiovascular events. Although deep learning (DL) models leveraging echocardiographic images from ultrasound are widely used for cardiac function analysis, their application to AHRE prediction remains unexplored. This study introduces a novel DL-based framework for automatic AHRE detection using echocardiograms. The approach encompasses left atrium (LA) segmentation, LA strain feature extraction, and AHRE classification. Data from 117 patients with cardiac implantable electronic devices undergoing echocardiography were analyzed, with 80% allocated to the development set and 20% to the test set. LA segmentation accuracy was quantified using the Dice coefficient, yielding scores of 0.923 for the LA cavity and 0.741 for the LA wall. For AHRE classification, metrics such as area under the curve (AUC), accuracy, sensitivity, and specificity were employed. A transformer-based model integrating patient characteristics demonstrated robust performance, achieving mean AUC of 0.815, accuracy of 0.809, sensitivity of 0.800, and specificity of 0.783 for a 24-h AHRE duration threshold. This framework represents a reliable tool for AHRE assessment and holds significant potential for early SCAF detection, enhancing clinical decision-making and patient outcomes.

**Graphical abstract:**

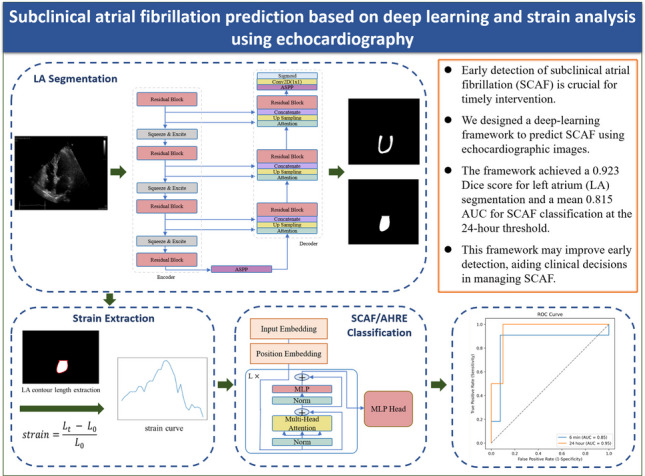

## Introduction

Atrial fibrillation (AF) is among the most common cardiac arrhythmias, associated with serious complications such as ischemic stroke, myocardial infarction, heart failure, and other cardiovascular conditions [1]. Atrial high-rate episodes (AHREs), also referred to as subclinical AF (SCAF), are defined as atrial rates ≥ 170 beats per minute lasting at least 5 min, detected through cardiac implantable electrical devices (CIEDs) or long-term rhythm monitoring [2]. AHREs, often identified incidentally, are linked to atrial cardiomyopathy and an elevated risk of progression to clinical AF and adverse cardiovascular outcomes [3]. Early detection of AHREs is, therefore, critical for timely intervention [4].

Cardiac imaging, particularly strain echocardiography via ultrasound, plays a pivotal role in assessing heart function, predicting risks, and guiding treatment for AHRE patients [5]. For example, initiating oral anticoagulants can reduce stroke risk or systemic embolism [6, 7], and early rhythm control may delay atrial arrhythmia progression, improving cardiovascular outcomes [8]. Among standard strain measurements—longitudinal, circumferential, and radial—longitudinal strain of the left atrium (LA) provides critical insights into heart function and early LA dysfunction [9]. This method is particularly valuable for detecting subtle changes, such as AF, as it helps identify early LA dysfunction [10]. Studies, such as those from the LOOP trial, have highlighted LA strain as an independent predictor of SCAF in at-risk individuals [11]. Despite the clinical significance of strain measurements in echocardiography, current approaches rely heavily on manual or semi-automated measurements using semi-automated software like EchoPAC™ [12], which are time-consuming, operator-dependent, and prone to variability.

Deep learning (DL)–based segmentation has advanced echocardiographic analysis by automatically identifying key regions of interest [13–15], including the left ventricle (LV) [16–19] and LA [20, 21], as well as the calculation of LV longitudinal strain [18, 19] and LA volume [21]. Most studies used UNet-based models [13, 21], while others explored optical flow–based networks [13, 18, 19], diffusion-based models [17], transformer-based models such as vision transformer [15], generative adversarial network (GAN)–based models [22], and hybrid models [16]. Although a range of approaches have been introduced to improve cardiac chamber segmentation with a few calculating cardiac functions such as LA volume and LV strain, the application of DL to predict AHREs using automatically extracted LA strain features remains unexplored. This represents a critical gap in leveraging DL for early detection and risk stratification of atrial arrhythmias.

This study introduces a novel DL framework that integrates LA segmentation, strain analysis, and patient clinical profiles to predict AHREs. By combining advanced imaging techniques with patient-specific data, this approach seeks to improve clinical decision-making and facilitate early identification of patients at risk, ultimately contributing to better prevention strategies and enhanced patient outcomes.

The rest of the paper is organized as follows: Sect. 2 presents the methodology, including the data and proposed framework. In Sect. 3, we demonstrate the effectiveness of our framework through experimental results and analyses, followed by a discussion in Sect. 4. Finally, Sect. 5 summarizes the key findings and implications of this work.

## Methods

### Data acquisition and preparation

#### Study participants

This retrospective study enrolled 117 patients (average age: 75.7 ± 10.0 years, 40% male) with CIEDs, treated between 2016 and 2020 at the National Yang Ming Chiao Tung University Hospital (NYCUH) in Yilan, Taiwan. Medical records and CIED interview data (conducted every 3–6 months) were reviewed, including patient comorbidities, CHA_2_DS_2_-VASc scores, and medication histories. This study adheres to the principles outlined in the Declaration of Helsinki. The study protocol was approved by the local Institutional Review Board (IRB), and the need for informed consent was waived. The details of the patient characteristics are summarized in Table 1. A total of ten patient characteristic features were extracted for developing AHRE classification models, as detailed later: age, sex, body mass index (BMI), body surface area (BSA), CHA_2_DS_2_-VASc score, myocardial infarction (MI), coronary artery disease (CAD), and comorbidities including thyroid disease, diabetes mellitus (DM), and hypertension (HTN).

**Table 1:** Baseline characteristics of the study population

	**Overall** **(*n* = 117)**	**AHRE < 6 min** **(*n* = 64)**	**6 min ≤ AHRE < 24 h** **(*n* = 40)**	**AHRE ≥ 24 h** **(*n* = 13)**	***p* value**
Age, years	75.7 ± 10.0	74.8 ± 11.2	77.4 ± 7.9	76.2 ± 10.0	0.353
Male, *n* (%)	47 (40)	30 (47)	11 (28)	6 (46)	0.313
Hypertension, *n* (%)	89 (76)	46 (72)	32 (80)	11 (85)	0.693
DM, *n* (%)	43 (37)	20 (31)	17 (43)	6 (46)	0.558
HF, *n* (%)	19 (16)	8 (13)	6 (15)	5 (38)	< 0.005
TIA/prior stroke, *n* (%)	6 (5)	5 (8)	1 (3)	0 (0)	0.610
Vascular disease, *n* (%)	27 (23)	17 (27)	8 (20)	2 (15)	0.280
Thyroid disease, *n* (%)	18 (15)	6 (9)	6 (15)	6 (46)	< 0.01
BMI (range)	24.6 ± 4.0	24.6 ± 4.1	23.9 ± 3.6	24.3 ± 4.2	0.435
CHA_2_DS_2_-VASc (range)	3.7 ± 1.4	3.6 ± 1.4	3.9 ± 1.2	4.2 ± 1.6	0.449
AHRE burden (%)	4.0 ± 12.8	0.06 ± 0.05	2.4 ± 6.1	26.7 ± 26.6	< 0.001
Medication, *n* (%)					
Oral anticoagulant, *n* (%)	49 (42)	13 (20)	25 (63)	11 (85)	< 0.001
Anti-arrhythmic drugs, *n* (%)	42 (36)	13 (20)	17 (43)	12 (92)	< 0.001
Beta-blocker, *n* (%)	56 (48)	33 (52)	14 (35)	9 (69)	0.505
ACEi/ARB, *n* (%)	59 (50)	34 (53)	19 (48)	6 (46)	0.800
Echocardiography					
LVEF (%)	54.6 ± 11.5	53.2 ± 12.9	56.2 ± 9.3	56.8 ± 9.0	0.307
LA Diam (mm)	41.6 ± 6.8	41.7 ± 7.3	39.9 ± 5.8	45.2 ± 5.5	< 0.05
E/E′ (ratio)	14.2 ± 5.2	14.7 ± 5.2	13.0 ± 5.5	14.6 ± 3.8	0.307
LA peak strain (%)	28.4 ± 10.6	31.1 ± 11.0	27.2 ± 8.3	19.9 ± 9.0	0.001

DM, diabetes mellitus; HF, heart failure; TIA, transient ischemic attack; BMI, body mass index; AHRE, atrial high-rate episode; ACEi, angiotensin-converting enzyme inhibitor; ARB, angiotensin receptor antagonist; LVEF, left ventricular ejection fraction; LA Diam, left atrial diameter measured by echocardiography in the parasternal short axis view; E/E′, E: early diastolic transmitral inflow velocities/E′: motion velocity of the septal mitral annulus in the early phase of diastole; LA, left atrium

#### Echocardiography dataset

Echocardiography was performed using the GE Vivid E95 system (GE Vingmed Ultrasound, Norway) with a 1.4–4.6 MHz transducer (M5Sc-D). All images were stored in the Digital Imaging and Communications in Medicine (DICOM) format and analyzed offline using the semi-automatic EchoPAC™ software (version 202, GE). The software delineated a ROI comprising six segments in each LA apical four-chamber (4 CH) view. The LA endocardial border was manually traced to measure LA peak strain.

**Fig. 1:**
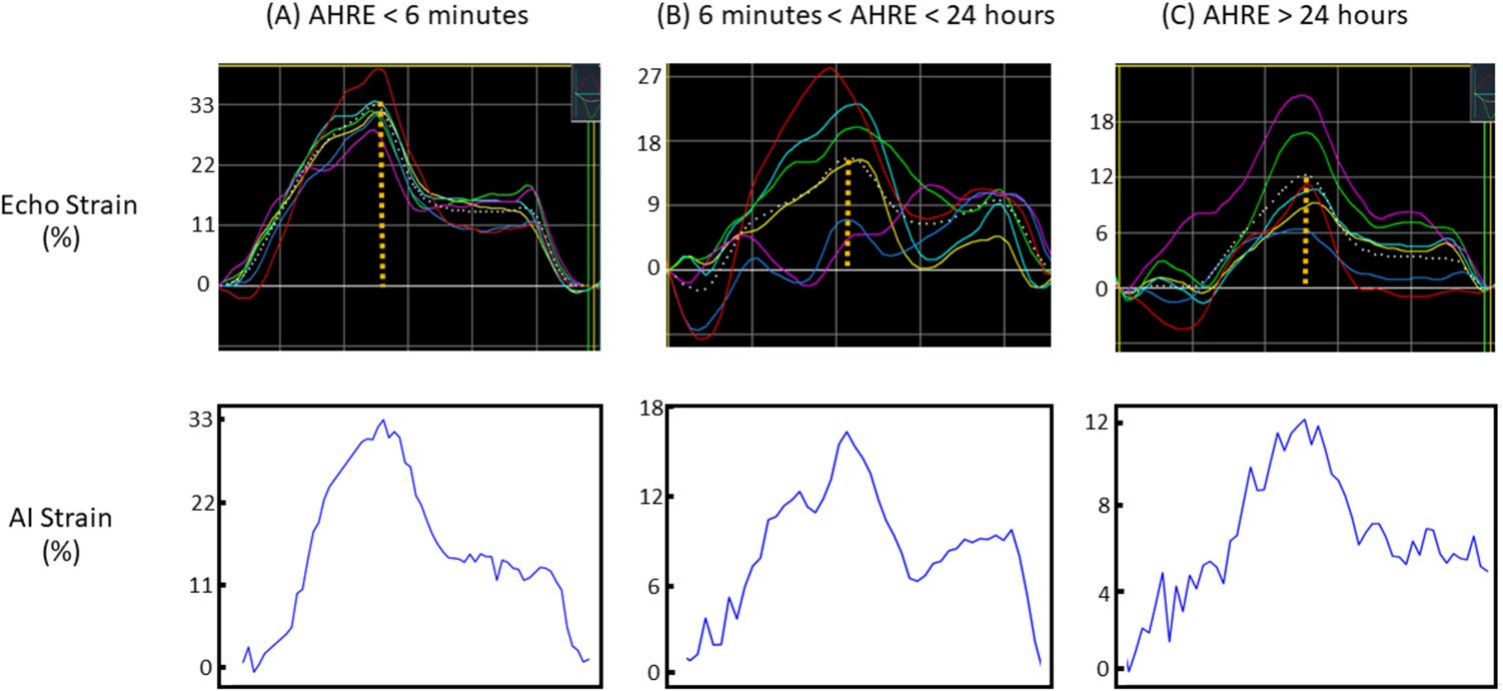
Representative cases demonstrating the process of strain measurement across varying durations and burdens of AHRE, using the semi-automatic EchoPAC™ software (upper panel) and the output from our deep learning (DL)-based framework (lower panel). The upper panels illustrate the apical four-chamber view in 2D echocardiography, showing six segmental strains derived from this view using the software. The peak strain (dashed line) is defined as the peak positive longitudinal strain relative to the zero reference at LV end-diastole. The lower panels present the corresponding strain values extracted by the proposed DL framework. A notable reduction in atrial peak strain is observed with increasing AHRE burden, particularly in panel (C), where AHRE durations exceed 24 h

Specialists from the Cardiology Department at NYCUH utilized the semi-automatic EchoPAC™ to mark the LA cavity and wall boundaries, which served as ground-truth annotations for segmentation models. The software initially attempts automatic contour detection of the LA, followed by manual adjustment of the endocardial border by our experienced specialists to ensure comprehensive coverage of the LA cavity while avoiding overlap with the pulmonary veins. The representative cases are shown in Fig. 1. The ground-truth LA strains were derived from calculations based on expert-annotated ground-truth annotations. For the classification tasks, patients were categorized based on the duration of AHREs with thresholds set at 6 min and 24 h [11].

**Fig. 2:**
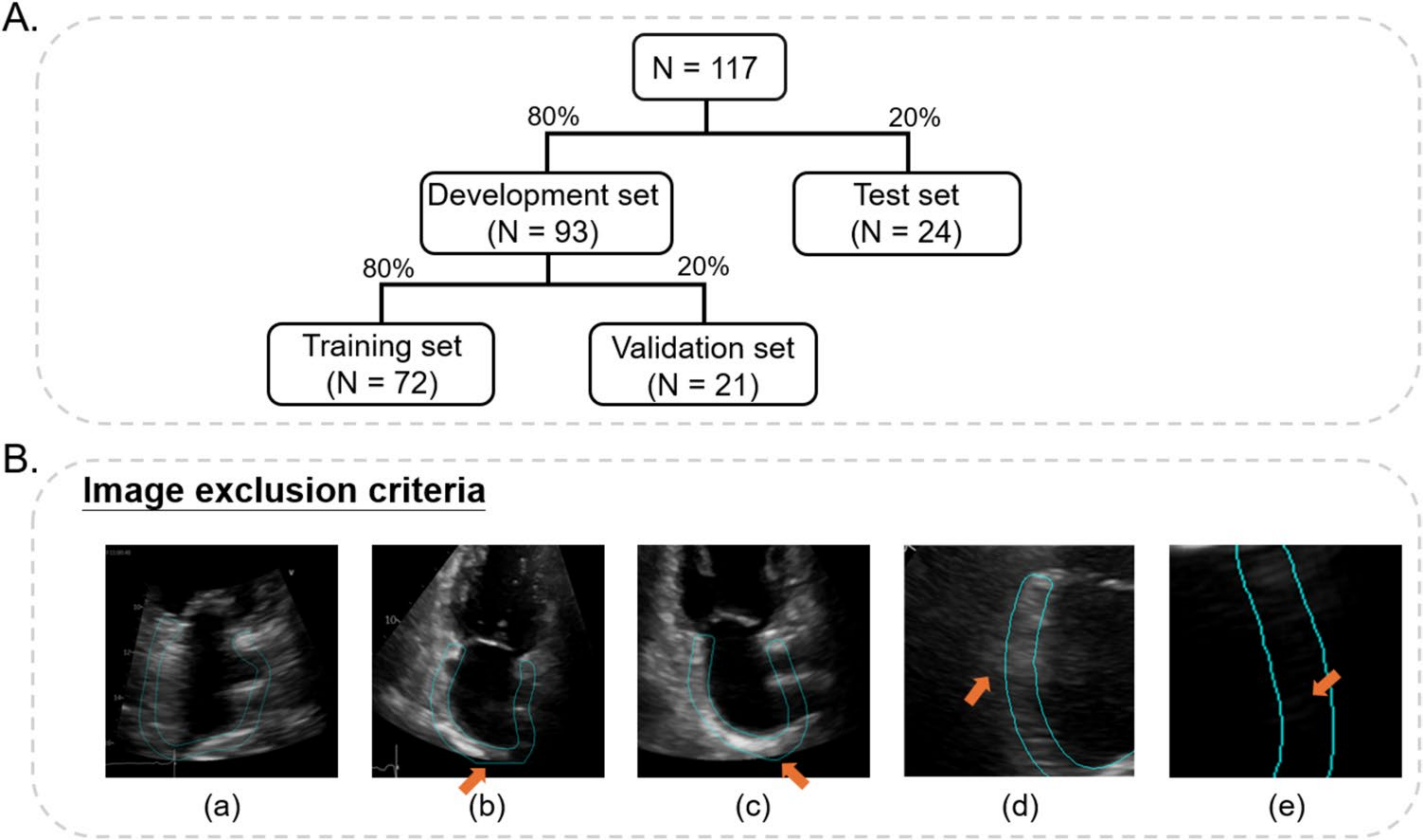
**A** Data subset sizes and **B** image exclusion criteria used to create a clean subset for developing the segmentation models. The exclusion criteria include the following: (a) images containing only a single chamber, introducing noise into the segmentation model; (b) cases where the bottom boundary of the ground-truth mask is truncated by the image boundary, indicated by an arrow pointing to a flat green line; (c) instances where the bottom boundary of the ground-truth mask extends beyond the echocardiographic area, highlighted by an arrow pointing to a small black region within the lower part of the green boundary; (d) images where the LA boundary appears blurred and lies outside the ground-truth mask, as indicated by an arrow; and (e) the presence of a significant black region within the ground-truth mask, likely caused by heart motion

The dataset was randomly stratified at the patient level into a development set (80%) and a test set (20%). The development set was further split into a training set (80%) and a validation set (20%) (Fig. 2A).

#### Clean subsets for segmentation

For LA segmentation, the original datasets were carefully reviewed to exclude images that could potentially mislead the segmentation model. As depicted in Fig. 2B, the exclusion criteria for creating clean subsets are as follows: (a) image with only one single chamber; (b) ground-truth mask truncated by the image boundary, as indicated by the arrow where a flat green line is present; (c) ground-truth mask extending beyond the echocardiographic area, as indicated by the arrow, where a small black region within the lower part of the green boundary; (d) blurred LA boundary that lies outside the ground-truth mask; (e) significant black region present within the ground-truth mask, likely caused by heart motion. A total of 1687 images from the training set and 449 images from the validation set were selected for training and validating the model. Notably, these subsets were used exclusively for the development of the segmentation models. The remaining components of the framework, including LA strain computation and AHRE classification, were developed using the entire training and validation datasets and tested on the full test dataset. This approach was adopted because the continuity of LA frames is essential for subsequently utilizing the calculated strains as input for the classification model, making it impractical to rely solely on the clean subset for these tasks.

### Framework

**Fig. 3:**
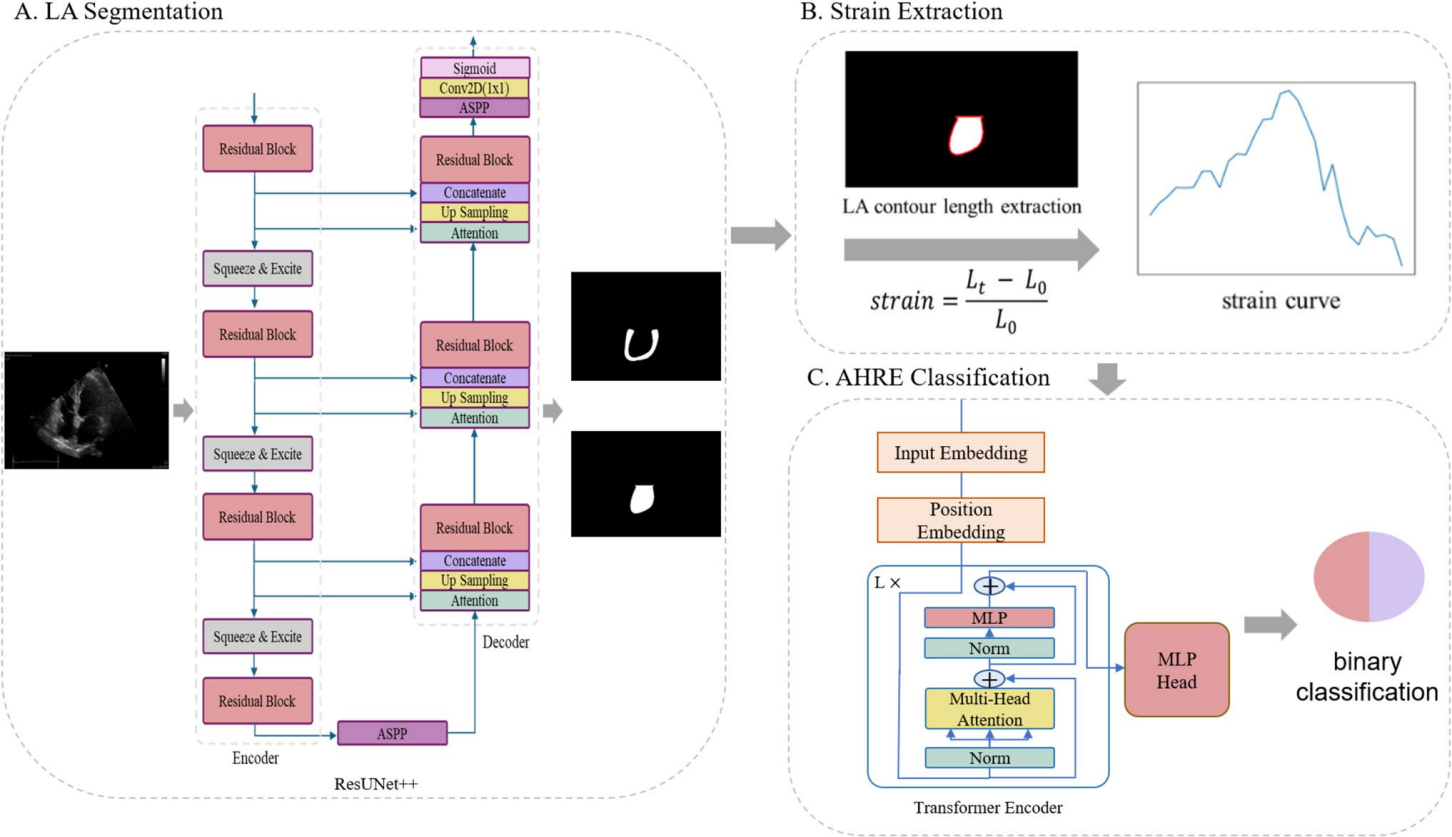
Overview of the proposed deep learning (DL) framework. **A** LA segmentation: a ResUNet + + model identifies and extracts the LA contour from echocardiographic images; **B** strain extraction: LA strain is derived using the contour lengths obtained from the segmentation results; and **C** AHRE classification: a transformer-based DL model utilizes the extracted strain features to classify patients into groups above or below the AHRE threshold

The overall framework of this study is presented in Fig. 3, which consists of LA segmentation, strain extraction, and AHRE duration classification.

#### LA segmentation

**Fig. 4:**
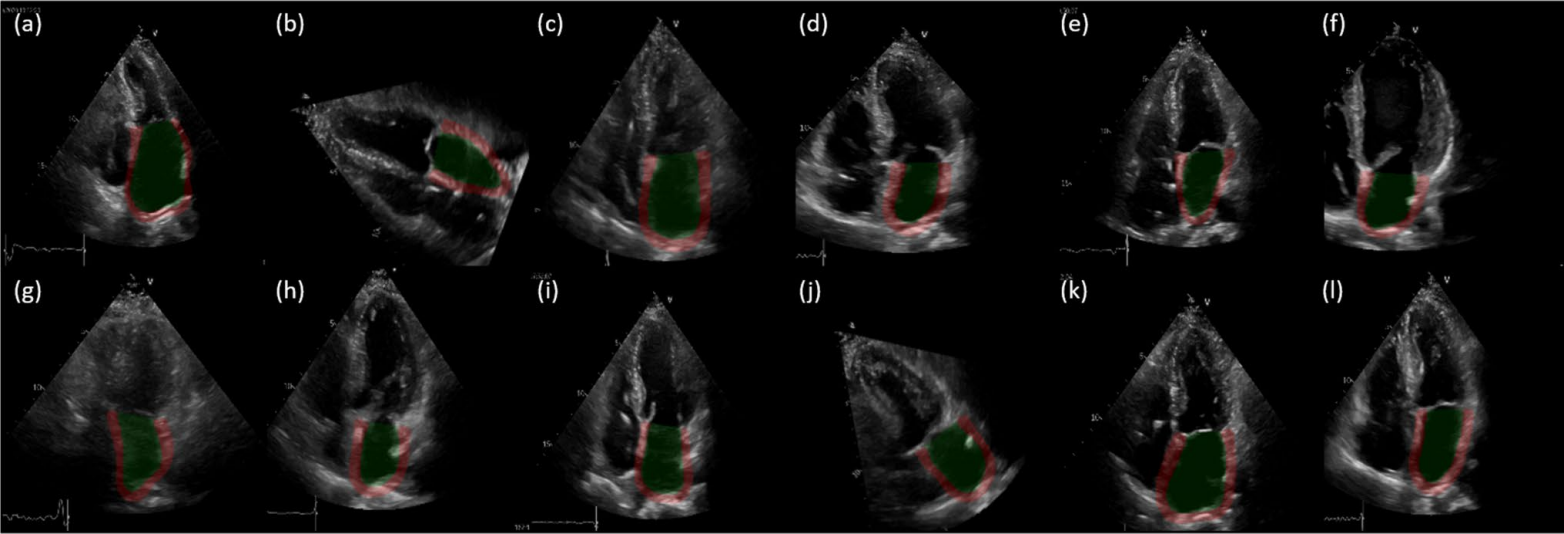
Randomly selected examples of data augmentation techniques, such as random cropping and rotation, applied during model training (**a**–**l**). The ground-truth left atrial (LA) cavity and wall are highlighted in green and red, respectively

For the LA segmentation component, we compared four segmentation models, including DeepLabv3 [23], UNet with a VGG 16 encoder (UNet16) [14], ResUNet++ [24, 25], and UNet with group normalization (UNetGN). Input images were resized to 224 × 224 pixels, and the models were trained to segment cavity and wall regions on echocardiographic images, as illustrated in Fig. 3A. Training utilized a combination of soft Dice loss [26] and standard cross-entropy loss. Data augmentation techniques, including random rotation and random cropping with the scale—the range of random crop sizes relative to the input size—set to (0.6, 1), were applied to improve model robustness. A few randomly selected examples of data augmentation generated by *TensorBoard* during training are presented in Fig. 4. Training was conducted on an NVIDIA RTX Titan 24GB GPU for 400 epochs, with a learning rate of 0.001, adjusted using a cosine annealing scheduler.

#### Strain extraction

LA peak strain represents the deformation of the atrial myocardium during early diastolic atrial relaxation and is defined as the percentage change in myocardial length over time relative to its initial length [27]. The strain at timestamp *t* is computed using the following formula:

$$strain=\frac{{L}_{t} - {L}_{0}}{{L}_{0}}$$

where ${L}_{t}$ denotes the contour length of the LA at each timestamp [13] and ${L}_{0}$ refers to the contour length measured at the base timestamp (the R peak of the ECG R-R interval). The peak strain is the maximum strain across all timestamps.

The endocardial length, defined as the contour length of the cavity, was used to represent the LA contour length (Fig. 3B). Given the thin LA wall and the higher segmentation accuracy achieved for the endocardium, the endocardial strain serves as a reliable surrogate for myocardial strain calculations. This approach is consistent with standardized practices in 2D speckle-tracking echocardiography, where the LA endocardial border is traced to assess myocardial deformation [28]. Additionally, since echocardiography uses ultrasound to generate real-time images of the heart, the number of frames in a recorded sequence can vary between patients. To maintain a uniform input size for our AHRE classifier, we standardized the selection to 30 frames using equidistant sampling. This number was determined based on the median frame count across all training samples.

#### AHRE classification

The extracted LA strain values, combined with patient characteristics, were used as input feature vectors for the AHRE classification model. A transformer model, illustrated in Fig. 3C, was trained for this purpose. The multi-head attention mechanism within the model effectively captures longitudinal relationships between strain measurements across different timestamps, enhancing prediction accuracy. To address the class imbalance in the data, a weighted binary cross-entropy (BCE) loss [29] was employed instead of the standard BCE loss [30], with weights adjusted according to the class imbalance ratios. The models were trained for 400 epochs with a learning rate of 0.001, utilizing a cosine annealing scheduler.

#### Evaluation metrics

The performance of the LA segmentation models was assessed using the Dice similarity coefficient (DSC), which quantifies the spatial overlap between predicted and ground-truth masks. The automatically extracted LA strains were validated against ground-truth strains, calculated from the ground-truth regions, using Bland–Altman (B-A) plots, which included bias and limits of agreement (LOA). For AHRE classification, evaluation metrics included the area under the receiver operating characteristic curve (AUC), accuracy, sensitivity, and specificity. Sensitivity and specificity were reported based on Youden’s *J* statistic.

## Results

### LA segmentation results

**Fig. 5:**
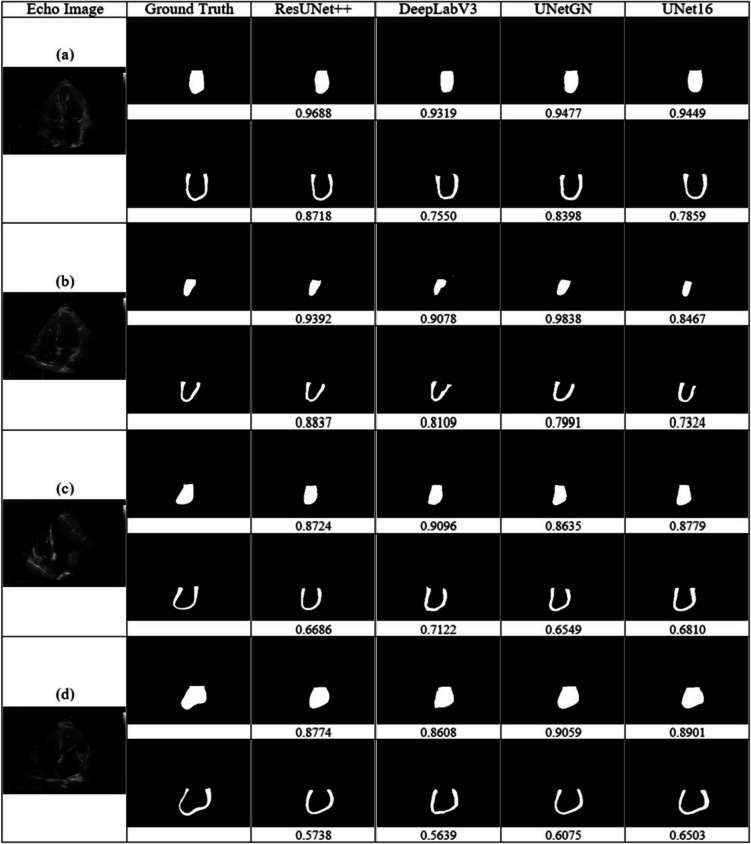
Examples of cavity and wall segmentation results from different models, compared with Dice scores calculated against the ground-truth masks. **a**,** b** Cases where ResUNet + + performs better, **c**, **d** cases where ResUNet + + performs the opposite

The models were initially compared using the clean validation subset, followed by an evaluation of the best-performing model on the test set. As summarized in Table 2, LA cavity segmentation demonstrated superior performance compared to LA wall segmentation. Among the evaluated models, ResUNet + + demonstrated superior performance on both test sets, achieving a DSC of 0.923 for LA cavity segmentation and 0.741 for wall segmentation on the clean test subset and a DSC of 0.902 for LA cavity segmentation and 0.693 for wall segmentation on the entire test set, outperforming DeepLabv3, UNet16, and UNetGN. Notably, the performance differences in cavity segmentation between ResUNet + + and other models were all statistically significant. Consequently, ResUNet + + was identified as the optimal model. Figure 5 presents example segmentation results for all models, with the DSC scores listed beneath each predicted mask. Figure 5a, b illustrates cases where ResUNet + + outperformed other models in both cavity and wall segmentation. In contrast, Fig. 5c, d depicts instances where ResUNet + + failed to accurately capture the LA contour, allowing other models to achieve better performance.

**Table 2:** Performance of LA cavity and LA wall segmentation on the clean validation subset and the entire test set

		ResUNet + +	DeepLabv3	*p* value	UNetGN	*p* value	UNet16	*p* value
Clean test subset	Cavity	**0.9230**	0.9144	< 0.001*	0.9205	< 0.05*	0.6076	< 0.001*
	Wall	**0.7414**	0.6427	< 0.001*	0.7365	0.0728	0.4869	< 0.001*
Entire test set	Cavity	**0.9016**	0.8710	< 0.001*	0.8809	< 0.001*	0.5850	< 0.001*
	Wall	**0.6931**	0.6344	0.6037	0.6808	0.7357	0.4646	0.3640

An independent *t*-test was employed to assess whether the performance differences between ResUNet + + and other models are statistically significant, with a *p* value threshold of < 0.05 indicating significance

Values in bold indicate the best performance

### LA peak strain extraction results

**Fig. 6:**
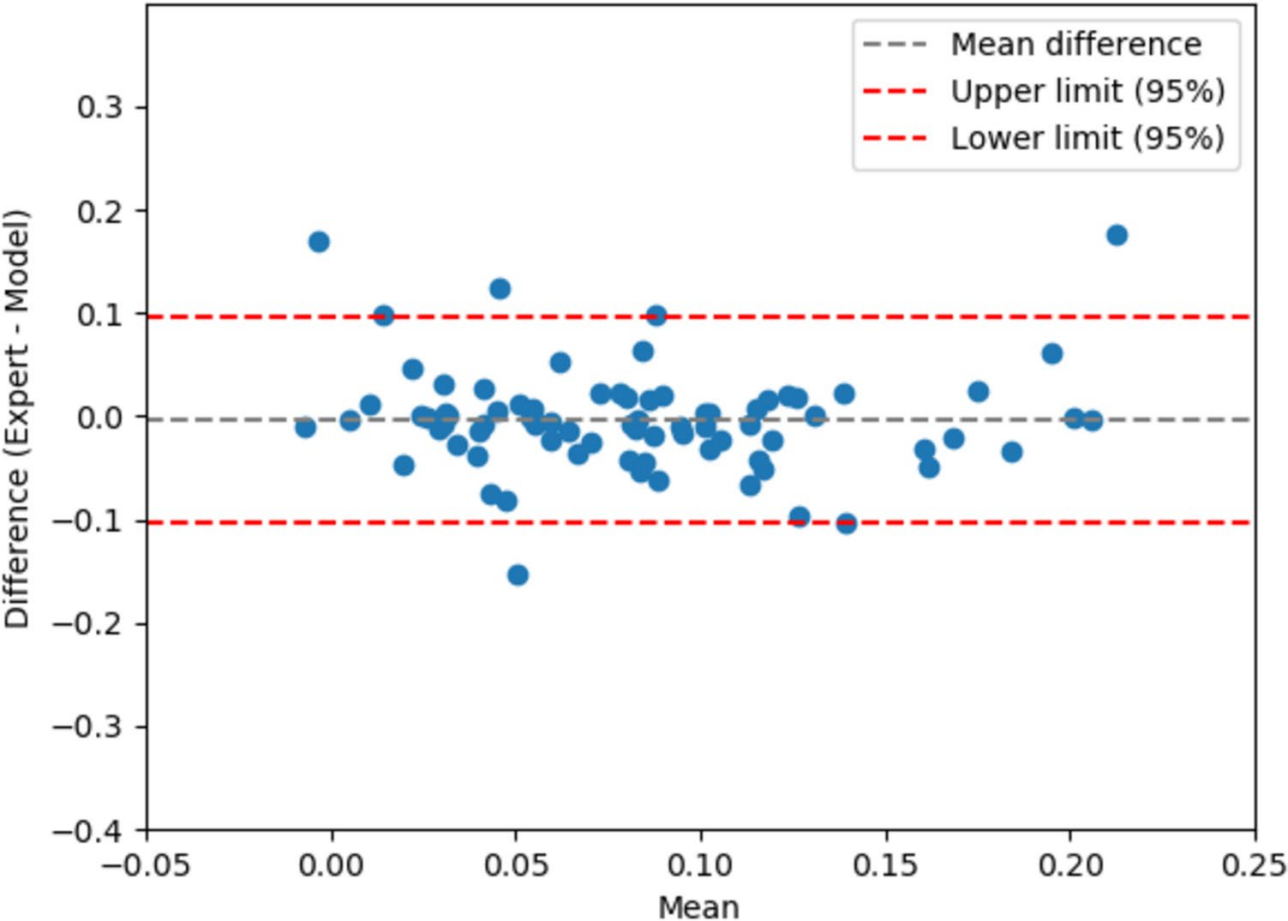
Comparisons between framework-extracted and expert-extracted peak LA strain using a B-A plot

The results of the B-A analysis show mean biases of −0.003 ± 0.05 for the peak strain. The 95% LOA ranges were −0.103 to 0.096 (Fig. 6). These results suggest that the automatically extracted peak strains from echocardiography are comparable to the ground truth.

### AHRE classification results

**Fig. 7:**
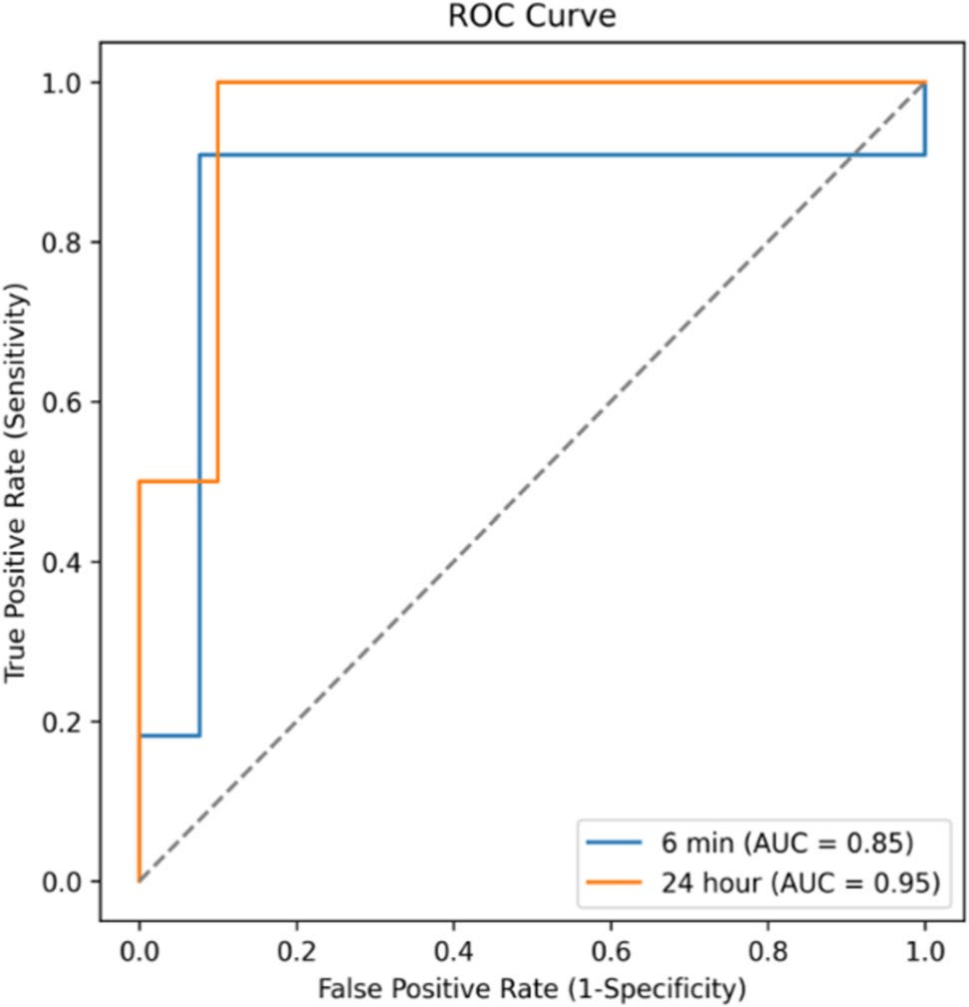
ROC curve of the transformer classification model using model-extracted strains and patient profiles as input features

In the AHRE classification stage, LA strains derived from the automatically detected LA contour lengths were used as features, alongside patient characteristics, to enhance model performance. As shown in Table 3, the model achieved an AUC of 0.853, an accuracy of 0.875, a sensitivity of 0.909, and a specificity of 0.846 at the 6-min AHRE duration threshold. At the 24-h threshold, the model exhibited promising performance on the test set, achieving an AUC of 0.950, an accuracy of 0.750, a sensitivity of 1.0, and a specificity of 0.700. Given the limited number of positive cases, we conducted a stratified fivefold cross-validation to further validate the promising results. As summarized in Table 3, the model achieved a mean AUC of 0.815 ± 0.14, an accuracy of 0.809 ± 0.09, a sensitivity of 0.800 ± 0.40, and a specificity of 0.783 ± 0.14. The ROC curves for AHRE classification are illustrated in Fig. 7.

**Table 3:** Performance of the transformer model combined with patient characteristics: AUC, accuracy, sensitivity, and specificity

AHREs	AUC	Accuracy	Sensitivity	Specificity
6-min	0.8532	0.8750	0.9091	0.8462
24-h	0.815 ± 0.14	0.809 ± 0.09	0.800 ± 0.40	0.783 ± 0.14

The classification results of the 24-h AHRE threshold are presented mean ± standard deviation using fivefold cross-validation as requested by the reviewer, due to the limited positive cases in the test set

## Discussion

This study introduces a novel DL-based framework for classifying AHREs using echocardiographic imaging and patient profiles. The framework demonstrates its overall efficacy by automating LA segmentation and strain extraction, achieving high precision in LA segmentation with the ResUNet + + model. Additionally, the transformer-based model demonstrated robust performance in classifying AHREs at both 6-min and 24-h thresholds, underscoring the framework’s potential for fully automated AHRE detection.

Model segmentation of the LA cavity was notably superior to that of the LA wall. This discrepancy can be attributed to the LA’s thin walls, posterior and superior positioning, and potential image quality limitations. Artifacts, noise, and the similar echogenicity of adjacent tissues, such as the liver, contribute to challenges in delineating the epicardial boundary [31]. Given these challenges, cavity segmentation results, which focus on endocardial boundary length, were utilized for strain analysis. Although computed tomography might serve as an alternative [32], it involves radiation and contrast exposure.

Imbalanced data is a common challenge in medical datasets, which can hinder accurate classification of minority class samples [30]. To address this, a weighted BCE loss was employed. As shown in Table 3, the framework achieved higher AUC and sensitivity for AHRE classification at the 24-h threshold, likely reflecting more pronounced LA disease in patients with extended AHRE durations. Moreover, its robust performance at the 6-min threshold (> 0.846 across metrics) underscores its reliability and versatility.

The ASSERT study reported a 2.5-fold increased stroke risk for device-detected subclinical AF (SCAF) lasting > 6 min, distinguishing true low-risk patients with episodes < 6 min [33]. Similarly, AHRE episodes > 24 h indicate high stroke risk [34], warranting anticoagulant therapy [1]. This DL framework effectively classifies patients by AHRE duration, aiding in precise risk stratification and early intervention.

Fully automated LA segmentation and strain analysis offer potential beyond AHRE classification, such as in evaluating diastolic function in heart failure (HF) patients. Studies have shown that DL models integrating LA strain with additional variables improve HF assessments [35]. In their study, LA function was still assessed using semi-automated software like EchoPAC. While our research focuses on SCAF, the fully automated LA imaging and strain analysis offer broader applicability in cardiovascular care across diverse patient populations.

Our study has several limitations. First, while strain imaging provides valuable insights, its accuracy can be affected by factors such as image quality, operator variability, and the specific strain parameters employed. Furthermore, the optimal strain thresholds for predicting AF risk may differ across populations and clinical settings [36]. Second, the moderate sample size presents a potential risk of overfitting in the deep learning model. However, this risk can be mitigated through strategies used in this study, such as data augmentation during training. Third, the analysis relied on a fixed number of 30 strain measurements to ensure consistency in the subsequent classification model training. While this approach ensures uniformity during the training process, it may not accommodate patients with fewer images. Future research should focus on developing methods to include patients with fewer images, enabling a more comprehensive analysis.

## Conclusions

Echocardiography is a non-invasive and well-established method for evaluating cardiac functions. In this study, we developed and evaluated a DL-based framework on echocardiograms to segment the LA for strain measurement and predict AHRE occurrence. The results showed that a transformer model trained on 4 CH view images and patient characteristics could accurately predict AHREs at a 24-h threshold with a mean AUC of 0.815, an accuracy of 0.805, a sensitivity of 0.800, and a specificity of 0.783. This finding demonstrates the potential of AI models for automatic LA assessment and AHRE prediction. This tool has the potential to substantially enhance the early detection of SCAF in clinical practice.
